# Density Structure of the Von Kármán Crater in the Northwestern South Pole-Aitken Basin: Initial Subsurface Interpretation of the Chang’E-4 Landing Site Region

**DOI:** 10.3390/s19204445

**Published:** 2019-10-14

**Authors:** Chikondi Chisenga, Jianguo Yan, Jiannan Zhao, Qingyun Deng, Jean-Pierre Barriot

**Affiliations:** 1 State Key Laboratory of Information Engineering in Surveying, Mapping and Remote Sensing (LIESMARS), Wuhan University, Box 129, Luoyu Road, Wuhan 430070, China; cchisenga@must.ac.mw (C.C.); dengqy@whu.edu.cn (Q.D.); jean-pierre.barriot@upf.pf (J.-P.B.); 2 Department of Earth Sciences, Ndata School of Climate and Earth Sciences, Malawi University of Science and Technology, Limbe P.O. Box 5196, Malawi; 3 State Key Laboratory of Geological Process and Mineral Resources, Planetary Science Institute, China University of Geosciences, Wuhan 430074, China; jnzhao@cug.edu.cn; 4 School of Environmental Studies, China University of Geosciences, Wuhan 430074, China; 5 Geodesy Observatory of Tahiti, BP 6570, Faa’a 98702, Tahiti, French Polynesia

**Keywords:** Von Kármán Crater, density structure, gravity inversion, Chang’E-4 mission

## Abstract

The Von Kármán Crater, within the South Pole-Aitken (SPA) Basin, is the landing site of China’s Chang’E-4 mission. To complement the in situ exploration mission and provide initial subsurface interpretation, we applied a 3D density inversion using the Gravity Recovery and Interior Laboratory (GRAIL) gravity data. We constrain our inversion method using known geological and geophysical lunar parameters to reduce the non-uniqueness associated with gravity inversion. The 3D density models reveal vertical and lateral density variations, 2600–3200 kg/m^3^, assigned to the changing porosity beneath the Von Kármán Crater. We also identify two mass excess anomalies in the crust with a steep density contrast of 150 kg/m^3^, which were suggested to have been caused by multiple impact cratering. The anomalies from recovered near surface density models, together with the gravity derivative maps extending to the lower crust, are consistent with surface geological manifestation of excavated mantle materials from remote sensing studies. Therefore, we suggest that the density distribution of the Von Kármán Crater indicates multiple episodes of impact cratering that resulted in formation and destruction of ancient craters, with crustal reworking and excavation of mantle materials.

## 1. Introduction

**Figure 1:**
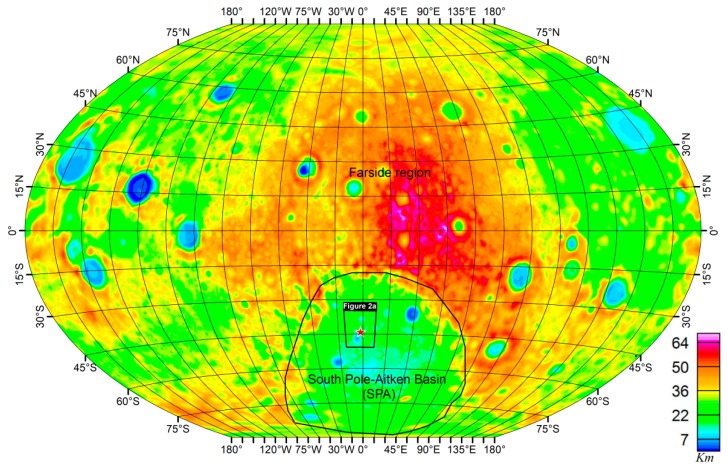
The crustal thickness map of the Moon, centered on the farside (Winkel tripel projection [7]). Also shown are the locations of the South Pole-Aitken Basin and the Von Kármán Crater. The black polygon shows the extent of the study area, which includes the Von Kármán Crater and surrounding craters. The red pentagram indicates the location of the Chang’E-4 landing site (177.59°E, 45.46°S; [11,12]).

The Von Kármán Crater is a pre-Nectarian crater with a diameter of ~186 km [1] within the largest known impact structure on the farside of the Moon, the South Pole-Aitken (SPA) Basin (Figure 1) [2]. The SPA is the oldest impact basin with a well-preserved impact history, which suggests that the earliest lunar history and processes related to impact cratering can be studied from it [3,4]. The SPA is subdivided into four distinct compositional zones [4], with the Von Kármán Crater situated within the Mg-Pyroxene Annulus zone. The Mg-Pyroxene Annulus contains the main materials that were excavated and melted by the SPA-forming event as indicated by its relatively uniform composition, great area, depth, and thickness. Subsequently, the Von Kármán Crater was filled with mare basalt around 3.6 Ga in the Imbrian period [2,5,6]. The Von Kármán Crater is further characterized by a very thin crust with an average of 10 km [7]. It also shows evidence of differentiated impact melts during its formation [3], low crater-floor elevation values with an average of 5926 m [2], relative to a mean lunar radius of 1737.4 km [8] and high Bouguer gravity values in the southern part [9]. The thin crust inferred from a gravity-derived crustal thickness model [7] implies that the mantle is uplifted in this region [7,9,10], with a possibility of upper mantle materials exposed on the surface [4]. These characteristics of the Von Kármán Crater make it a good candidate for subsurface studies using gravity data, as density distribution beneath the Von Kármán Crater could complement the geological and spectral studies.

The China’s Chang’E-4 lunar exploration spacecraft successfully landed on the Moon in January 2019 [11], and started the first in-situ exploration of the farside of the Moon. The landing region was located in the southern part of the Von Kármán Crater, within the mare basalt region [12]. A detailed geological and spectral analysis in the Von Kármán Crater indicates compositional and mineralogical differences for basaltic rocks compared to the nearside of the Moon. The surface materials in this region are characterized by a low titanium content, few mafic minerals and iron-depleted mare basalts [2,13], which forms a basis for further interpretation of the in-situ exploration. Some previous studies, however, have indicated that mantle materials are possibly exposed in the SPA region [4], while some studies cast doubt on such occurrences [13,14,15]. However, the first published results from the Chang’E-4 mission [16] show that the surface of the Von Kármán basin is characterized by mafic components that are dominated by low-Ca pyroxene (LCP) and olivine, with a very small amount of high-Ca pyroxene (HCP), which suggest a deep-seated, upper mantle origin [17]. Despite the presence of mare and other basaltic materials on the landing site of the Change’E-4 in the Von Kármán Crater [4], it is suggested that these excavated mantle materials originated from the Finsen Crater in the northeast [13,16], with a possibility that they originated from the base of the differentiated melt sheet [18]. These studies, however, do not consider the subsurface extent of the surficial manifestation of the mare basalt and possible mantle excavated materials. Our study therefore correlates the surface basaltic materials with the subsurface density distribution. We investigate the geophysical characteristics of the subsurface structures for the Von Kármán Crater using GRAIL gravity data. The presence of small-scale features revealed by the GRAIL gravity data [9,19] was correlated to the near surface materials, its porosity and compositional differences within the Von Kármán Crater [19,20,21,22]. We then identify the existence and possible extent of differentiation of the SPA melt sheet [18,23] and the possible subsurface extension of the surficial basaltic material [2,13] based on density models using density inversion method. The approach gives a 3D subsurface characteristic of the Von Kármán Crater that furthers our understanding of the origin of the basaltic surface materials. Therefore, it complements the geological and spectral analysis of the Chang’E-4 in-situ acquired data, which subsequently echoes the surface anomalies of possible mantle sources.

## 2. Materials and Methods

### 2.1. GRAIL Gravity Data

The Gravity Recovery and Interior Laboratory (GRAIL) mission significantly improved the gravity resolution and accuracy of the Moon, adding value to the existing lunar gravity models from Luna Prospector, Kaguya and other existing data [24]. The first phase of the data collection, also called the Primary Mission, of GRAIL mapped the gravity field of the Moon beginning from March 1st, 2012 up to May 29th, 2012, flying at a mean altitude of 55 km [25,26]. This was followed up by the second phase, also called the extended mission, that mapped the Moon starting on August 30th, 2012 and ending on December 14th, 2012, collecting data at a lower mean altitude of 23 km [25,26]. The GRAIL mission thus produced a lunar gravity field of unprecedented quality with a high spatial resolution expanded to spherical harmonic for different degrees and orders, e.g., 660, 900, and 1200 [9,27,28]. Such improvement has increased the spatial resolution by a factor of 3 to 4, with the RMS power of the GRAIL data error ~5 orders of magnitude smaller than the RMS power error in previous models [27]. High resolution data have the ability to resolve smaller anomalies on the Moon [19], which improved the results obtained from previous gravity models. 

**Figure 2:**
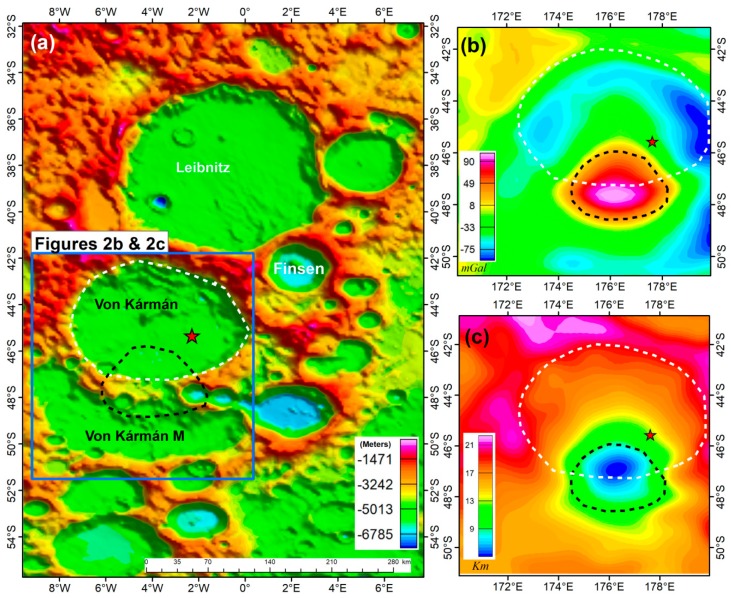
Study area. (**a**) Topographic map of the broader Von Kármán region and the surrounding craters, farside centred, after Barker et al. [8]. The Von Kármán Crater is enclosed by several other impact craters, namely, the Leibnitz crater in the north, the Finsen crater in the northeast and the Von Kármán M in the south. Also shown are the extent of our gravity inversion (Blue polygon), the boundary of the Von Kármán raised rim (White-dashed polygon) and the extent of high gravity region (Black-dashed polygon; Figure 2b). (**b**) Lunar Bouguer anomaly from the GRGM1200A gravity model available for the spherical harmonic degree and order 1200 [9], truncated between 6–450 degrees, nearside centred, after Goossens et al. [9]; (**c**) The crustal thickness map of the Von Kármán Crater, nearside centred, after Wieczorek et al. [7]. The red pentagram shows the location of the Chang’E-4 landing site (177.59°E, 45.46°S; [11,12]).

To achieve the objective of the study, we used higher spatial resolution (~4.5 km) GRAIL gravity data expanded to a spherical harmonics degree and order 1200, extracted from the GRGM1200A model [9]. The Bouguer anomaly data were calculated using SHTOOLS [29,30,31] at a height of 10 km with a Moon reference radius of 1738 km. The bulk density of the South Pole-Aitken, in which the Von Kármán Crater is located, is ~2880 kg/m^3^ [19]. The density value is higher than the average crustal density of 2550 kg/m^3^ for the feldspathic highlands crust [7]. It is also noticed that the typical SPA interior is more mafic than feldspathic material [32], which is consistent with the use of the higher average density value of ~2880 kg/m^3^ [18]. Thus, we used this assumed density value for the South Pole-Aitken Basin in the gravity data calculation to obtain the Bouguer anomaly. The gravity data were then truncated between degrees 6 to 450 to avoid long-wavelength data from the deep mantle and noise that is contained in short-wavelength signals as a result of orbit parallel stripping at higher altitude (Figure 2). The resulting filtered data highlight mid- to short-wavelength crustal sources required for the subsurface analysis [25].

### 2.2. Gravity Derivative Calculation

In order to characterize the relationship between the surficial and near surface basaltic materials and the deep-seated structures, the Bouguer gravity data were subjected to first-order vertical derivative and tilt angle derivative calculations [33]. The calculations enhance the small-scale anomalies of the crustal structures [34,35]. These techniques are effective as the basaltic materials have different density values than the abundant and wide-spread surrounding feldspathic crustal materials [36,37,38,39,40]. In this scenario, the first vertical derivative estimates the rate of change of density values of basaltic materials in the vertical direction. The tilt-angle derivative, however, is very effective as it normalizes all the density changes, such that subtle and less evident small-scale features in the horizontal and vertical directions are well enhanced. Thus, we used the derivative results to reveal the surface and near-surface basaltic materials. We also compared the derivative results with the small-scale features recovered from 3D density inversion, which could indicate that the inversion was successfully applied.

### 2.3. Three Dimension (3D) Density Inversion

#### 2.3.1. Inversion Algorithm

In this section, we describe the mathematical description of the inversion algorithm, which is applied in spherical approximation by using spherical prisms, called tesseroids. This density inversion algorithm has been tested on synthetic data and applied to the Moon [41,42,43,44]. It is based on the Li and Oldenburg [45,46] depth weighting algorithm. The basic formula for the depth weighting inverse problem for the algorithm is given by Equation (1):

(1) $$\emptyset \left(m\right)=\emptyset_{d}+\mu \emptyset_{m}=\|W_{d}{\left(Gm-d^{obs})\right\|}_{2}^{2}+\mu \|W_{m}{\left(m-m_{ref})\right\|}_{2\;\;}^{2\;\;}$$

In Equation (1), $\emptyset_{d}$ is a measure of data misfit. It is represented using a 2-norm measure [45,46] where $W_{d}$ is a data weighing matrix with a diagonal elements of $1/\sigma_{i}$ in which σ is the standard deviation of the *i^th^* datum. G is the kernel function that denotes the relationship between the geological model (*m*) and the observed data ($d^{obs}$). The model objective function ($\emptyset_{m}$), also called a model norm, measures the smoothness of the model. $W_{m}$ is also a weighting matrix for the model objective function that defines the closeness and smallness between the recovered model (*m*) and the reference model ($m_{ref}$). Thus, the objective function ∅(m) for the inversion combined the model objective function and the data misfit, and is controlled by a regularization parameter (μ). A regularization parameter [47] basically balances up the model objective function and data misfit. The best fit value of μ lies at the corner of the L_curve for a plot of the model norm against data misfit [48,49,50]. The original objective function [45,46] was designed to work in a Cartesian coordinate system. This poses a difficulty when applied to a large area with noticeable surface curvature on the Moon due to its small radius. To overcome this limitation, Liang et al. [44] extended the model objective function within the depth weighting algorithm for application in a spherical coordinate system [44]. They modified the depth weighting function in the algorithm using uniform prism cells to re-scalable prism cells along the radial direction from the surface going downwards designed for the spherical coordinate system (scs).

The solution of the inversion problem is obtained by minimizing the objective function (Equation (1)) following Equation (2). This equation is solved as the conjugate gradient (CG) matrix with an iterative algorithm, 

(2) $$\left[G^{T}W_{d}^{T}W_{d}G+\mu W_{m}^{T}W_{m}\right]m=G^{T}W_{d}^{T}W_{d}d^{obs}+\mu W_{m}^{T}W_{m}m_{ref}$$

In Equation (2) $G^{T}$, $W_{d}^{T}$ and $W_{m}^{T}$ are transpose matrices for the G, $W_{d}$ and $W_{m}$ that were described in Equation (1). We also include the Lagrangian multiplier [51,52] method introduced by Zhang et al. [41] within the model objective function to fit the constraints information. This increases the reliability of the inversion results. For instance, the penalty factor compels the recovered model to be more reliable, whilst the geological constraints improve the inversion results [43]. The resulting extended formulae (Equation (3)) for this constrained inversion algorithm is described by Zhang et al. [43] and is given by:

(3) $$\begin{array}{l} \left[G^{T}W_{d}^{T}W_{d}G+\mu W_{m}^{T}W_{m}+\frac{1}{2}M\left(F_{1}+F_{2}+F_{3}\right)\right]m\\ =G^{T}W_{d}^{T}W_{d}d^{obs}+\mu W_{m}^{T}W_{m}m_{ref}-F_{0}\lambda_{0}^{T}+F_{1}\lambda_{1}^{T}+F_{2}\lambda_{2}^{T}\\ +\frac{1}{2}M\left(F_{0}m_{0}+F_{1}m_{1}+F_{2}m_{2}\right)+\frac{1}{2}M\left(F_{1}Z_{1}^{2}-F_{2}Z_{2}^{2}\right) \end{array}$$

In Equation (3), the diagonal of the matrix *F_i_* denotes the index of the constrained information in each divided rectangle cell, M is the penalty factor, λ represents Lagrangian multipliers, and $Z_{i}$ is the slack variable of the *i*th cell, where *i* is the index number for each cell that represents defined geological/geophysical bounds constraints.

#### 2.3.2. Implementation

The dataset and the inversion space were prepared using the 3D mesh as illustrated in Table 1. The spatial resolution of the data was increased from ~4.5 km [9] to ~6 km (or 0.2°), given that 1° is equivalent to ~30.2 km on the Moon. The depth of the 3D mesh was constrained to 22 km, which represents the deepest point of the crust-mantle boundary in our study area [7]. The depth extent is consistent with the calculated Bouguer gravity data, which are mostly characterized by signals from the lunar crust. Then, the distance in the radial direction was discretized into cell sizes of 0.5 km. The final 3D mesh had 98, 400 data cells (50 cells in the longitudinal direction × 48 cells in the latitudinal direction × 41 cells in the radial direction) that occupied a physical volume space of 26, 299, 735 km^3^ (302 km × 295.95 km × 22 km). The geophysical bound constraints, representing density values, within each cell in the 3D mesh were set to −580 kg/m^3^ and 520 kg/m^3^. These values represent the geophysical/geological upper and lower bound constraints, based on the crustal densities, relative to the average density of the South Pole-Aitken Basin. Normally, lunar crust densities vary between 2300 kg/m^3^ and 2900 kg/m^3^ with an average of 2550 kg/m^3^ [7]. A 3D mesh with a flat base extending to 22 km could include the mantle, since the greater part of the study area has crustal thicknesses less than 22 km, with the thinnest crust at 5 km. Furthermore, the region is known to have occurrences of basalts that have higher density values [2,6,16,23]. Thus, we extended the density variation to 3300 kg/m^3^; hence, the values of the upper bound exceed the difference between the average density value of the Aitkin Basin, ~2880 kg/m^3^, and the average lunar crustal density, ~2550 kg/m^3^. As a result, the background density for each cell was set to zero (0) kg/m^3^ that represents the 2880 kg/m^3^ in absolute density terms, to obtain the density contrast within the upper and lower bound constraints.

**Figure 3:**
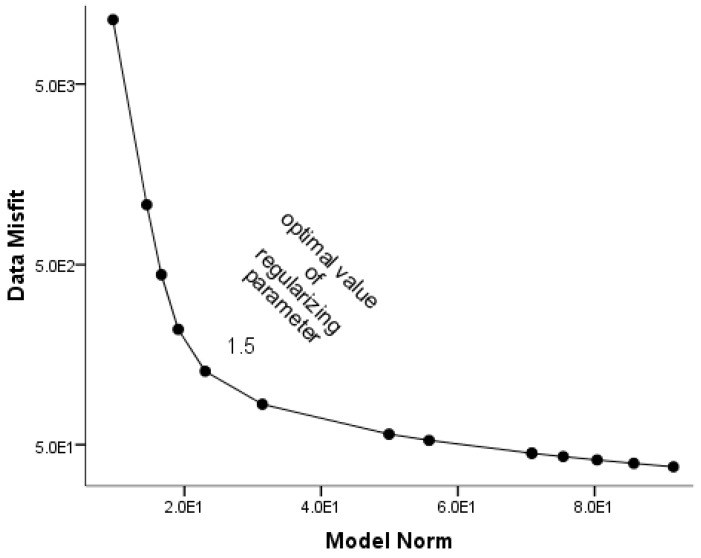
L_curve solution for data misfit ($\phi_{d}$) against model objective function ($\phi_{m}$ ). The plot, called the L_curve method, indicates the optimal value of regularization parameter as a value that lies at the corner of the L_curve.

**Figure A1:**
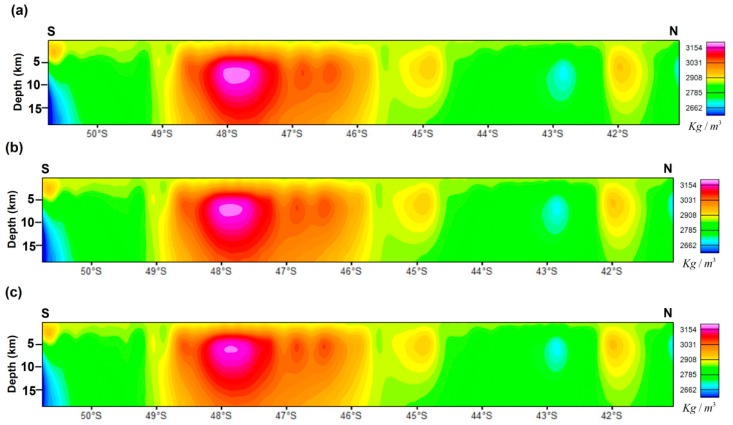

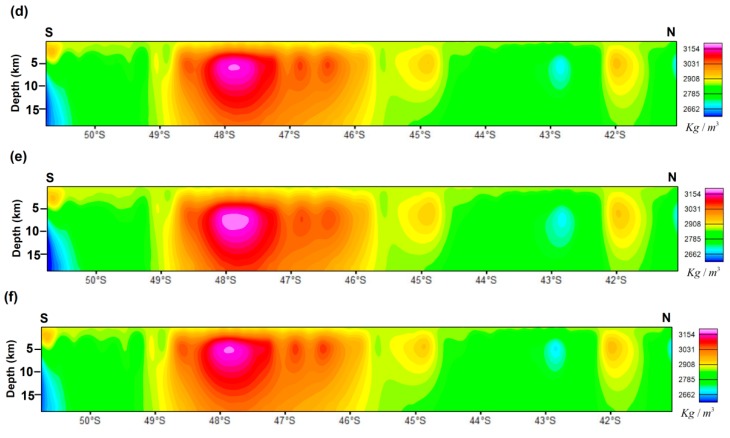
Some of the models that were recovered from changing the exponent of the Tikhonov parameter during 3D inversion for (**a**) 0.2, (**b**) 0.3, (**c**) 0.4, (**d**) 0.5, (**e**) 1 and (**f**) 1.5.

**Figure A2:**
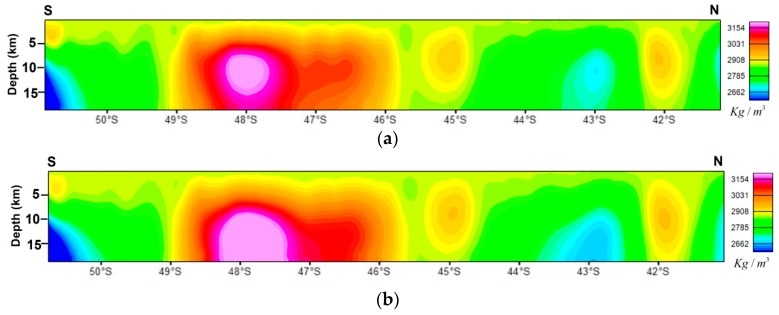

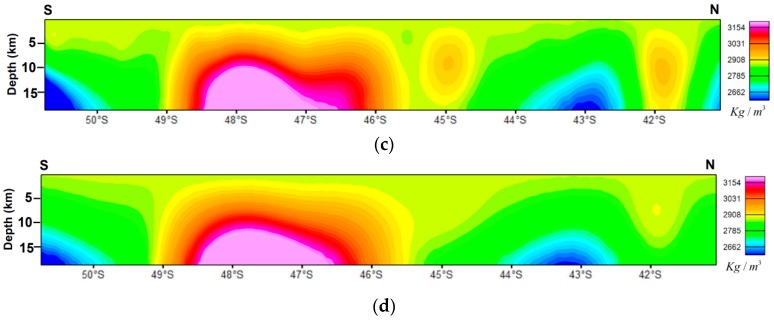
Some of the models that were recovered from changing the exponent of the Tikhonov parameter during 3D inversion for (**a**) 2, (**b**) 2.5, (**c**) 3 and (**d**) 4.

During the inversion, some parameters, i.e., roughness factor, length scale, depth weighting parameter, were kept constant (Table 2), based on previously accepted studies and extensive test results [41,45,46]. However, a penalty factor was assigned to a small value of 1.0 × 10^−6^ (with an increment of 2) and iteratively increased until the penalty factor fit the constrained information [41]. The same was applied to the criteria for terminating the iteration by defining the convergence values for the inversion and conjugate gradient (CG) method (Table 2). Each of the iterative results was forward modeled in spherical approximation by numerically solving the Gauss-Legendre quadrature (GLQ) integration problem [53], thereby finding an optimal solution that minimizes the data misfit and model objective function. Despite this parameter control, the gravity inversion procedure is still an ill-posed problem that suffers from non-uniqueness, which produces multiple solutions. We controlled this and identify a best fit solution by inverting the data with 14 different values of the regularization parameter (Table 2; Figure 3). The 14 values were obtained after experimenting with a number of ranges to produce 14 different models (Figure A1 and Figure A2). The plotted L_curve for the 14 regularizing parameters (Figure 3) identifies the value of 1.5 as a best fit regularization parameter, which enabled a construction of a desirable density model. This model was used for geophysical interpretation of the Von Kármán Crater as discussed in Section 3. 

## 3. Results

**Figure 4:**
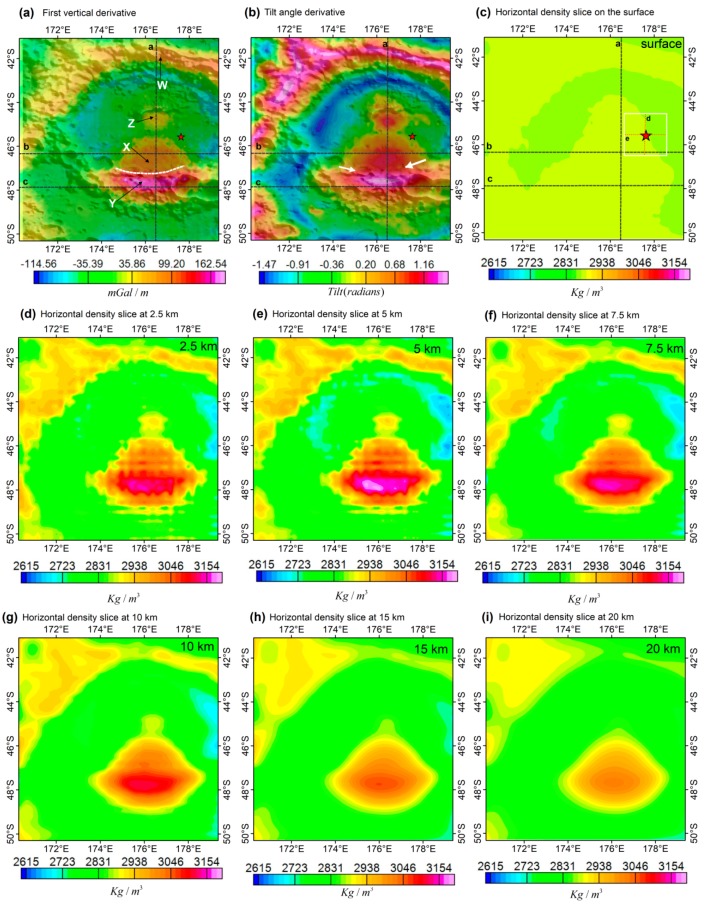
Derivative calculations results draped on a hill-shade topographic map, a-b; (**a**) First vertical derivative. The letters X, Y, Z and W represent anomalous areas in this region. The dashed-white line in Figure 4a represents a possible boundary that separates the high-density region from a relatively low-density region. and (**b**) Tilt angle derivative; and the horizontal density slices c-i, at the depth of (**c**) surface, (**d**) 2.5 km, (**e**) 5 km, (**f**) 7.5 km, (**g**) 10 km, (**h**) 15 km and (**i**) 20 km. The three black lines (a-c) in Figure 4a–c represent the location of the vertical cross-section shown in Figure 5, and the red dashed (d and e) in Figure 4c lines represents the location of the vertical cross-section across the landing site.

**Figure 5:**
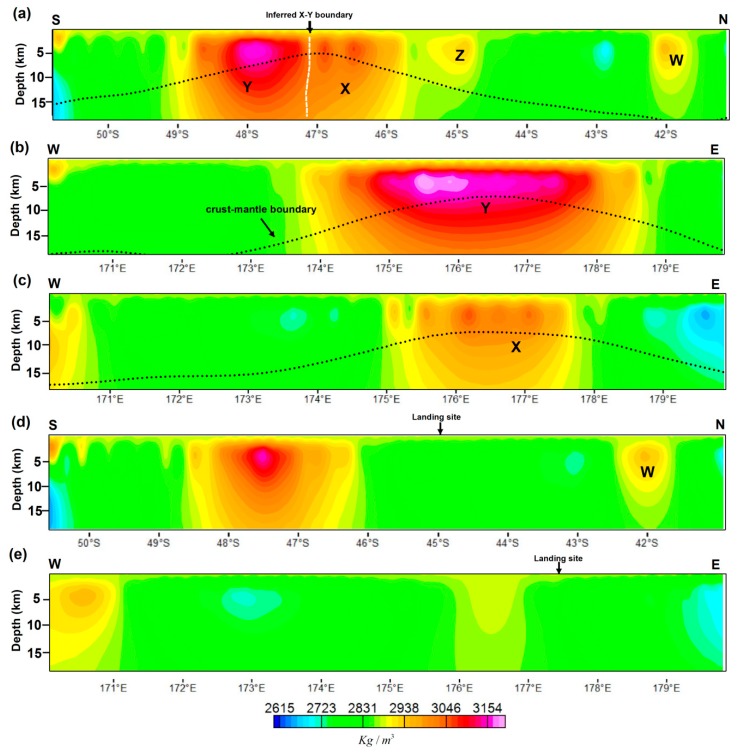
Vertical cross-section maps for the Von Kármán Crater. A vertical cross section, (**a**–**c**), reveals the subsurface density distribution of locations shown in Figure 4 of the same letters, with a vertical exaggeration of 2. A vertical cross-section, (**d**,**e**), indicates the subsurface structure across the landing site (177.59°E, 45.46°S; [11,12]). The black dotted line indicates the crust-mantle boundary from model 2 of Wieczorek et al. [7].

The recovered 3D density structures beneath the Von Kármán Crater indicate the presence of both lateral and horizontal density variations that range from 2600 kg/m^3^ to 3200 kg/m^3^ (Figure 4 and Figure 5). We also noticed that the small-scale density anomalies are confined to the upper 8 km of the crust. The anomalies observed on the density slice up to the depth of 5 km can also be seen in the first vertical derivative and the tilt angle derivative (Figure 4a,b), which validates our inversion results that resolved the small-scale anomalies. The inversion results also show short-wavelength artifacts up to the depth of 7.5 km (Figure 4c–f) that were introduced in the results primarily due to the use of a radial cell size of 0.5 km. The crust-mantle boundary is generally thin in the study area, with high-density anomalies, >3000 kg/m^3^, located below this boundary. The density values increase with an increase in crustal depth beneath the high gravity regions. However, in other areas, especially the immediate area just below the crust-mantle boundary of the Von Kármán Crater, the density values decrease with an increase in crustal depth. The upper 2 km indicates a relatively low-density value of ~2900 kg/m^3^ and conversely the region between 2 km and 10 km reveals a higher density value of greater than 3000 kg/m^3^, which could be due to presence of buried and thick near surface basaltic materials [2,13] that overlay the feldspathic crust [54,55]. The crustal model number 2 of Wieczorek et al. [7] from GRAIL gravity data was plotted on the vertical cross-section as a depth reference. The density models do not correlate with the crustal thickness as the two are constructed from different levels of gravity anomalies, since crustal thickness modeling is subjected to heavy filtering that removes most of the gravity signal to attain model stability [7,29].

Figure 4 and Figure 5 reveal both shallow and deep subsurface density structures of the Von Kármán Crater. The shallow subsurface structures are clearly visible even from the derivative maps (Figure 4a,b), which are also consistent with the recovered density model (Figure 4c–f). The northern rim of the Von Kármán Crater, labeled W, reveals an anomalous region (Figure 4a,b), with a density value of ~2900 kg/m^3^ that extends to the depth of ~10 km (Figure 5a). This area is flanked by low gravity regions within the base of the elevated region, noticed in both the derivative maps and the density slices up to the depth of 7.5 km. The southern rim of the Von Kármán Crater, however, reveals the highest density values, ~2900 kg/m^3^ on the surface (Figure 5) that go up to 3150 kg/m^3^ on the crust-mantle boundary. Below the crust-mantle boundary, the density decreases to ~3000 kg/m^3^. The crust is noticeably thin in this region [7]. This anomaly forms an elliptic and elongated E-W trending feature along the elevated areas, apparently squeezed between the Von Kármán and the Von Kármán M Craters.

The high-density subsurface structures (labeled Y) show an obvious annulus of a low-density anomaly, which is more visible on the gravity anomaly than on density models. Unlike other regions with a similar high gravity center surrounded by an annulus of low gravity features [56,57,58], the annulus in this region transcends two impact craters, the Von Kármán and the Von Kármán M. The low-density annulus are consistent with the thicker crust surrounding the high-density central part (Figure 2 and Figure 3), which also transcend these two craters. Adjacent to this body, an extension of the high-density anomaly of ~2950 kg/m^3^ is located within a flat crater floor, also noticed on the topographic map (Figure 4a–c). The derivative maps (Figure 4a,b) reveal a slightly low-gravity region between these two bodies (white dashed line on Figure 4a and pointed by two white arrows on Figure 4b). The striking dissimilarity between these two anomalies, low-density northern part, labeled X, and relatively higher-density southern part, labeled Y, suggests that these two anomalies were possibly formed as a single body but underwent different post formation conditions. The possible boundary of these two anomalies is placed on the ~2900 kg/m^3^ region (dashed white line on Figure 4a) with the subsurface density structures, indicating two regions (Figure 4c–e, Figure 5b,c).

The high-density anomaly ends on the edge of the Chang’E-4 landing site (red star on Figure 4), which suggests that the landing site is directly above the feldspathic crust, overlain by considerable amount of mare basalt [13]. These characteristics are also revealed by the vertical cross-section on the landing site (Figure 5d,e). The densities are slightly higher than the underlying crust, ~2800 kg/m^3^. Another high-density anomaly, labeled Z, is located in the middle of the Von Kármán Crater. The anomaly indicates possible buried materials of the crater’s central peak within the crust, with a density value of ~2850 kg/m^3^. The high density associated with this anomaly is entirely located above the crust-mantle boundary (Figure 5a). The density values of this anomaly are slightly comparable with the values of the northern rim anomaly, labeled W, but lower than the southern anomalies, labeled X and Y. 

## 4. Discussion

### 4.1. 3D Density Model and Evolution for the Von Kármán Basin

The 3D gravity inversion has revealed a prominent mass excess on the southern rim of the Von Kármán Crater. The subsurface configuration of this feature, with a bowl-shaped anomaly, thin crust and a high-density region beneath the surface characterizes a buried impact basin [59,60,61]. The Bouguer gravity anomaly in this region (~85 mGal) is relatively small to indicate uplifted mantle materials. Instead, it could be derived from the filled basalts in the impact crater [61]. However, the noticeable different subsurface structural configurations, with high-density asymmetry, could indicate an increase in density towards the southern part of the high-density region, which suggests a possible decrease in porosity or an increase in the mafic content [7,19,21]. We hypothesize that the structures were initially formed by a single impact cratering event. This is further evidenced by an annulus of low gravity values that surrounds the high-density anomaly. We also suggest that the high-density region predates the Von Kármán Crater, possibly formed during the formation of the Von Kármán M Crater. Stratigraphic sequences and dating techniques indicate that the Von Kármán Crater postdates and is superimposed on the Von Kármán M Crater, obscuring the crater morphology of the Von Kármán M Crater [13]. Thus, we discuss the formation and evolution of the mass excess on the southern rim of the Von Kármán Crater in relation to the Von Kármán M Crater.

We investigate the possible mode of formation for the mass excess related to our proposed multiple impact cratering. We propose that the mass excess beneath the Von Kármán and Von Kármán M Craters was formed by three impact events as a result of multiple impact cratering. The first major impact event, that occurred more than 4 Ga years ago, in the multiple cratering scenario created the SPA that eventually excavated the lower crust and probably the upper mantle, resulting in a very thin crust region [7,23]. Then, this event was followed by another impact event that created the Von Kármán M Crater, similar to the formation of an ancient mascon [56,57,62]. This event happened between 4 Ga and 3.97 Ga years ago, as dating techniques indicate that the Von Kármán M Crater predates the Von Kármán Crater [13]. However, the mantle materials did not push the crust to attain isostatic stability in this region, as noticed from the lack of the excessive high-density anomaly beneath the crust-mantle boundary. This impact event probably excavated an already thinned crust forming a bowl-shaped geometry and possibly further exposing mantle materials in the Von Kármán M Crater. The Von Kármán M Crater was subsequently filled by mare basalts that created a buried impact basin [59,61] with high-density and high gravity anomalies labeled X and Y. Finally, a third impact event reworked the Von Kármán M Crater and created the Von Kármán Crater. In the process, the existing high-density region beneath the Von Kármán M Crater was probably pushed southwards or thermally reworked during the impact. The other explanation is that the northern part of the mass excess (labeled X) was excavated by the impact event that created the Von Kármán Crater while the southern part (labeled Y) returned its basaltic thickness. This could explain the density asymmetry of the body. The presence of a central peak, labeled Z on Figure 4, suggests that the high-density anomalies, X and Y, predate the Von Kármán Crater and that the central peak is associated with the formation of Von Kármán Crater. Thus, the high-density region was created during later stages of multiple cratering and was not excavated during the formation of SPA. The Von Kármán Crater was also later filled by mare basalts, since multiple volcanic lava flows in the Von Kármán Crater region have been identified by remote sensing techniques to have occurred in different time periods, e.g., ~3.75 Ga [6], ~3.35 Ga [63], 3.15 Ga [6].

### 4.2. Implication of the Von Kármán Density Structure for the Chang’E-4 Mission

The outstanding research question in the formation of SPA hinges on the possibility that the upper mantle was excavated and brought to the surface. The Chang’E-4 mission explored this possibility with initial interpretation confirming the presence of mantle materials from the presence of olivine and LCP [16]. Li et al. [16] and Qiao et al. [13] suggested that the mantle materials, with the presence of olivine and LCP, originated from the Finsen Crater and were transported to the Von Kármán Crater. Our inversion results, however, reveal that the mass excess anomalies (Y and X on Figure 4) were created as the result of possible multiple impact cratering that reshaped and concentrated the anomaly in the southern end of the Von Kármán Crater. The mass excess anomaly labeled X has an apparent correlation with both surface mineralogy and mare volcanism [4,13]. The floor of the Von Kármán Crater is characterized by mare basalts in the southern part and feldspathic materials in the northern part. This is evidenced in the recovered density models, with >2900 kg/m^3^ values localized in the southern part. Qiao et al. [13] also identified an additional possible underlying basaltic layer, beneath the mare basalt layer, at a depth up to ~30–90 m, characterized by slightly higher iron and titanium contents.

In Section 4.1, we indicated that the second impact event that created the Von Kármán M Crater excavated a thinned crust that probably contained upper mantle and lower crust materials. The mare basalts that were deposited in this region could have buried the mantle materials, which were eventually excavated by the third impact event that created the Von Kármán Crater. Since it is shown that the materials of the Chang’E-4 landing site have a strong resemblance to the mantle originated mafic central peak of the Finsen Crater, we suggest that the impact cratering that formed the SPA basin excavated mantle materials. Then, re-excavation by the impact forming the Von Kármán Crater exposed the mantle materials. This is evidenced by the absence of mantle materials in region Y, and high correlation of basaltic materials with the presence of olivine and LCP with region X [16]. Possibly, the mare basalts mixed with mantle materials, indicating a relationship between the mantle materials on the floor of the Von Kármán Crater and the Finsen Crater. We thus suggest that the impact forming the Von Kármán Crater contributed somewhat to the secondary excavation of mantle materials that were initially excavated by events that formed the SPA and were buried by the mare basalts that occurred after the impact event that created the Von Kármán M Crater.

## 5. Conclusions

We performed a 3-D inversion of the GRAIL gravity data to produce a density model of the Von Kármán Crater. Our results reveal that the region is underlain by a mass excess anomaly beneath the Von Kármán M Crater with a density of ~3200 kg/m^3^, on the southern rim of the Von Kármán basin. A relatively high-density mass excess anomaly with a density value of up to 3100 kg/m^3^ connects to the first anomaly in the northern part. Basaltic rocks covering the Von Kármán basin show an extended body on the surface, with the northern part of the Von Kármán basin indicating a more feldspathic composition. We suggest that this was as the result of the buried mare basalts that created a buried impact basin in the Von Kármán M Crater. The inversion results are consistent with the spectral and geological results from remote sensing and gravity derivative data. The occurrence of high-density materials that extend to the lower crust correlates with excavated mantle materials observed on the floor of the Von Kármán basin, suggesting that the impact cratering could have brought upper mantle materials to the surface. We thus infer that the evolution of the Von Kármán basin was due to multiple episodes of impact cratering that resulted in crustal reworking and secondary excavation of mantle materials. 

## Figures and Tables

**Table 1:** The 3D mesh and dataset for inversion.

Region	Inversion Range	Model		Data	
		Grid Size	Grid Number	Data Size	Data Number
Von Kármán	Longitude	0.2°	50	0.2°	50
	Latitude	0.2°	48	0.2°	48
	Depth	0–2 km		0–50 km	
	Radial direction	0.5 km	41	0.5 km	41

**Table 2:** Inversion parameters.

Final Parameters for the Density Inversion		Determining the Optimal Value of the Regularization Parameter				
Parameter	Value	No	Tikhonov Parameter Exponent	Tikhonov Parameter	Data Misfit Value	Model Norm Value
Roughness factor	2, 2, 2	1	4.00	10,000.00	22714.7	9.55
Length scale	1 × 10^−10^	2	3.00	1000.00	2146.65	14.49
Depth weighting parameter	2	3	2.50	316.20	879.65	16.63
Penalty factor	1 × 10^−6^	4	2.00	100.00	437.87	19.09
Increase number for penalty factor	2	5	1.50	31.62	256.41	23.03
Convergence threshold of the inversion	1 × 10^−4^	6	1.00	10.00	167.94	31.41
Maximum iteration number of the inversion	1000	7	0.50	3.16	114.40	49.92
Convergence threshold of the CG method	1 × 10^−8^	8	0.00	1.00	75.13	91.54
Maximum iteration number of the CG method	500	9	0.05	1.12	78.55	85.74
		10	0.10	1.26	82.09	80.37
		11	0.15	1.41	85.75	75.41
		12	0.20	1.59	89.51	70.83
		13	0.30	1.59	89.51	70.83
		14	0.40	2.51	105.65	55.79
